# Omega-3 Index and Anti-Arrhythmic Potential of Omega-3 PUFAs

**DOI:** 10.3390/nu9111191

**Published:** 2017-10-30

**Authors:** Narcis Tribulova, Barbara Szeiffova Bacova, Tamara Egan Benova, Vladimir Knezl, Miroslav Barancik, Jan Slezak

**Affiliations:** 1 Institute for Heart Research, Slovak Academy of Sciences, Dubravska cesta 9, P.O. Box 104, 84005 Bratislava, Slovakia; barbara.bacova@savba.sk (B.S.B.); tamara.benova@savba.sk (T.E.B.); miroslav.barancik@savba.sk (M.B.); jan.slezak@savba.sk (J.S.); 2 Institute of Experimental Pharmacology and Toxicology, Slovak Academy of Sciences, Dubravska cesta 9, 84104 Bratislava, Slovakia; vladimir.knezl@savba.sk

**Keywords:** omega-3 PUFAs, omega-3 index, connexin-43, atrial fibrillation, ventricular fibrillation

## Abstract

Omega-3 polyunsaturated fatty acids (PUFAs), namely eicosapentaenoic acid (EPA) and docosahexaenoic acid (DHA) are permanent subjects of interest in relation to the protection of cardiovascular health and the prevention of the incidence of both ventricular and atrial arrhythmias. The purpose of this updated review is to focus on the novel cellular and molecular effects of omega-3 PUFAs, in the context of the mechanisms and factors involved in the development of cardiac arrhythmias; to provide results of the most recent studies on the omega-3 PUFA anti-arrhythmic efficacy and to discuss the lack of the benefit in relation to omega-3 PUFA status. The evidence is in the favor of omega-3 PUFA acute and long-term treatment, perhaps with mitochondria-targeted antioxidants. However, for a more objective evaluation of the anti-arrhythmic potential of omega-3 PUFAs in clinical trials, it is necessary to monitor the basal pre-interventional omega-3 status of individuals, i.e., red blood cell content, omega-3 index and free plasma levels. In the view of evidence-based medicine, it seems to be crucial to aim to establish new approaches in the prevention of cardiac arrhythmias and associated morbidity and mortality that comes with these conditions.

## 1. Introduction

Omega-3 polyunsaturated fatty acids (PUFAs), eicosapentaenoic acid (EPA) and docosahexaenoic acid (DHA) are permanent subjects of interest in relation to the protection of cardiovascular health, supported by an increasing number of both experimental and clinical studies, published in the last decade. Many of them indicate the benefit of acute or long-term omega-3 PUFA treatment, which is based on the prevention or reduction of systemic and heart disease-related adverse effects and consequently, of sudden cardiac death (SCD). Purified ethyl ester omega-3 PUFAs, rich in EPA and DHA (e.g., Omacor, Avaza, Vascazen, Omazen, etc.), cannot be considered nutritional adjuncts, only because they exert drug-like properties. Compared to drugs, undesired side effects of omega-3 PUFAs are rare. This may be a result of a sudden increase in endogenous exogenous myocardial tissue levels of omega-3 PUFA levels in the specific (e.g., ischemia-related) conditions, as noted in this article.

Experimental studies included in this review have demonstrated numerous mechanisms, by which circulating and incorporated omega-3 PUFAs may act at the cellular and molecular levels, including genetic and epigenetic modulations. Accordingly, omega-3 PUFAs are powerful modulators of various biological processes, such as cardiac function, in normal and pathological conditions.

The efficacy of omega-3 PUFA intake is manifested more obviously in the prevention and treatment of coronary heart disease (CHD) than in the prevention or treatment of life-threatening arrhythmias. An analysis of the literature in the PubMed database from the last decade suggests that, similar to previous decades, some recent studies and, in particular clinical trials, did not demonstrate significant anti-arrhythmic benefits from omega-3 PUFAs treatment. However, these studies and meta-analyses did not address in detail the limitations of their studies (e.g., small number and heterogeneity of participants, drug management, etc.) and there was also a lack of explanation of why the benefit was not observed. Unfortunately, many of these studies did not monitor basal omega-3 PUFA status, i.e., omega-3 index and circulating free omega-3 PUFA levels prior to interventions. These important issues should be addressed to allow progress in this field and for future perspectives [1].

Due to the fact that current anti-arrhythmic invasive and drug therapy is not fully efficient and that it is associated with complications, there is substantial effort aimed at preventing the occurrence and/or recurrence of life-threatening arrhythmias. These include ventricular fibrillation (VF), which increases the risk for SCD in patients without an implantable cardioverter defibrillator (ICD) and atrial fibrillation (AF), the most frequent type of arrhythmia in clinical conditions, which jeopardizes the life of patients, due to thromboembolism and stroke. A literature search based on articles in the PubMed database over the last decade was performed and data from the most recent studies are presented in this review, unless they were not reviewed previously. The purpose of this review is to provide an updated view on the role of omega-3 PUFAs in the protection of the heart against life-threatening arrhythmias and to outline future perspectives.

## 2. Most Recent Data on the Omega-3 Index (O3I)

The omega-3 index (O3I) (calculated as the proportion of EPA and DHA in red blood cell membranes) has already been suggested as a cardiovascular disease (CVD) risk factor in 2004 and was recently reviewed [1], indicating a target range of 8–12%. It is assumed that this range may decrease the risk for the development of arrhythmias. In this context, it is interesting to provide the latest data on the O3I in various populations, reported over the past few years and some data that has not been reviewed previously. However, it should be emphasized that the value of the O3I is difficult to compare in the studies if the standardization of methodology, using a well-established analytical procedure (see method HS-Omega-3 Index^®^, Omegametrix GmbH, Martinsried, Germany), was not performed [1].

The O3I, assessed in 503 French subjects aged 35–64 years, was 6.02 ± 1.7% and the inverse association with socio-economic status was explained by an insufficient seafood intake [2]. The O3I in Australians, aged about 76 years-old was significantly higher in females (12.99 ± 3.3%) compared to males, (11.92 ± 2.6%) and was inversely associated with plasma triglycerides and the total cholesterol/high-density lipoprotein (HDL) cholesterol ratio and was positively associated with HDL-cholesterol in all subjects [3]. A cross-sectional study [4] found that participants (aged about 75 years-old) with a low O3I had worse performance-based test results for physical function, than people with a high O3I (absolute data not shown)*.* Intermediate (>4% to <8%) or high (>8%) levels of O3I were related to higher HDL-cholesterol levels in white Canadians, but not in South Asians living in Canada [5]. Compared to white Canadians, the O3I may not be a good predictive risk factor for the prevalence of CVD and diabetes in South Asians, at the age of 20–79 years.

O3I was determined in 1301 adolescents, aged 13–15 years, and assessed in association with diet, lifestyle and socio-economic factors as well as cardiovascular and metabolic risk factors [6]. The mean O3I was 4.90 ± 1.04% (range 1.41–8.42%). When compared with categories identified in adults, 15.6% of adolescents were in the high-risk category (index < 4%). O3I was positively associated with dietary intakes of EPA and DHA, proteins, omega-3 fats and fish and wholegrain food groups, and negatively associated with the intake of soft drinks and crisps. The predictability of the O3I for the risk of CVD later in life warrants further investigation in the adolescent population.

Several studies have shown that the omega-3 PUFA status of women in Western countries is low. The nationwide cross-sectional German VitaMinFemin study included 446 women (40–60 years old) and showed that the average O3I of the total study population was 5.49 ± 1.17% [7]. In this study, the results were affected by factors, such as age and smoking. Compared to the target range of 8–12%, 62.8% of women had an O3I of 4–6%. Women taking hormonal contraceptives showed lower EPA levels and higher ratios of DHA/EPA which suggests an increased risk of CVD. It might be interesting to explore the influence of estrogen on omega-3 PUFA status.

The proportions of omega-3 PUFAs in erythrocyte membranes, plasma fatty acids and concentrations of plasma omega-3 PUFA-derived lipid mediators were significantly lower in vegans, aged 40–70. This was associated with lower day-time heart rate variability (that may predict SCD) in vegans compared to non-vegans [8].

A global survey of omega-3 PUFAs [9] revealed that regions with high EPA + DHA blood levels (>8%) included the Sea of Japan, Scandinavia, and areas with indigenous populations or populations not fully adapted to Western food habits. Also of interest is the fact that very low blood levels (≤4%) were observed in North America, Central and South America, Europe, the Middle East, South-East Asia, and Africa [9].

Interestingly, people with schizophrenia and depression have low O3I [10]. The average O3I was shown to be 3.95%, compared to an estimated 5% in the Australian population. This unfavorable omega-3 profile in people with a mental illness suggests a higher risk of CVD.

It has been reported that short-term (ten weeks) intake of high-dose (2.7 g/day) DHA increases the O3I more than a high dose of EPA in men and women (of reproductive age) with abdominal obesity and subclinical inflammation [11]. Moreover, the difference between DHA and EPA in increasing the O3I had a tendency to be higher in men than women. This suggests a potential sex-dependent difference in the response to dietary omega-3 PUFAs.

Heart diseases or systemic diseases significantly affect the target values of O3I. This value was 4.8% in patients with CHD who were reported to be low fish consumers [12]. A subset of patients participating in the Gruppo Italiano per lo Studio della Sopravvivenza nell'Infarto Miocardico (GISSI)-Heart Failure (HF) study treated with omega-3 PUFAs (1 g/day) exhibited an increase in the O3I from 4.8 ± 1.7% to 6.7 ± 1.9% [13]. This may explain the observed benefits with respect to cardiovascular events, despite the fact that the target levels were not achieved. However, such an increase seems to be insufficient to suppress the incidence of malignant arrhythmias significantly, according to the results from the sub-study of GISSI-HF on patients with ICD [14]. The causes of the reduced levels of the omega-3 PUFAs in HF remain unresolved. Most likely they are associated with alterations in cardiac metabolism and structural remodeling. The severity of myocardial structural alterations may, in part, explain the divergent findings [14].

An earlier study, involving ICD patients with HF (New York Heart Association classification II and III) showed that the baseline O3I was significantly elevated, as compared to control subjects (5.12 ± 0.87% vs. 4.24 ± 0.96%) [15]. The O3I was the only independent predictor for ventricular arrhythmias for up to 9 months. After 12 months, a reduced ejection fraction was an additional risk predictor for SCD.

Patients with AF had a significantly lower percentage of erythrocyte membranes containing omega-3 PUFAs than individuals without AF (2.8 ± 1.8% vs. 5.3 ± 1.1%) [16]. Low levels of myocardial tissue omega-3 PUFAs have been shown to increase the risk of VF during the acute ischemic phase of acute myocardial infarction (AMI) [17]. The median value of the O3I in VF cases was 4.88%, as compared to 6.08% in controls. After adjustment for age, sex, ejection fraction, high-sensitivity C reactive protein, use of beta-blockers, differences in infarct characteristics and previous angina pectoris, a 1% increase in the O3I was associated with a 48% reduction in risk of VF. The O3I remained stable following a sudden cardiac arrest (mean values 4.59% and 6.48%) and predicted the risk of VF [18].

Of note is the fact that a correlation between the O3I and bleeding during AMI was studied in 1523 patients from 24 United States of America (US) centers [19]. The rates of serious bleeding and mild bleeding were identified in patients with low (<4%), intermediate (4–8%) and high (>8%) omega-3 indices. There were no differences in bleeding across the O3I categories, suggesting that bleeding should not preclude the use of omega-3 PUFAs supplements when clinically indicated.

In a cross-sectional sub-study from the BALANCE Program, 364 patients with established CVD were enrolled for the determination of plasma fatty acid levels to estimate their intake and association with inflammatory biomarkers [20]. Results showed that PUFAs were inversely associated with C-reactive protein levels and with interleukin-1b.

Finally, our recent experimental study [21] demonstrated a lower O3I in both male and female spontaneously-hypertensive rats compared to non-hypertensive rats (1.0% vs. 2.2%). This rat strain mimics essential hypertension in humans and it is prone to developing malignant arrhythmias [22,23]. While the supplementation of rats with omega-3 PUFAs increased the O3I in hypertensive and non-hypertensive animals (up to 2.4% and 5.2%, respectively) [21], it was associated with reduced VF incidence in hypertensive rats and VF inducibility in the non-hypertensive animals [22,23].

Taken together, it appears that the O3I may be a good candidate as a biomarker for assessing the risk of cardiac undesirable events, including the occurrence of VF and AF. The O3I indicates that the incorporated cardiac tissue omega-3 PUFAs content increases, due to long-term omega-3 PUFAs supplementation. In addition, circulating or free omega-3 PUFAs exert acute beneficial effects, such as anti-inflammatory, antioxidant and anti-arrhythmic effects. However, available data suggest that the anti-arrhythmic (therapeutic) window for circulating omega-3 PUFAs is narrow. Thus, relatively high levels of free omega-3 PUFAs (e.g., due to infusion) may not always be associated with protection of the acutely injured heart against arrhythmias. Nevertheless, monitoring of both the O3I and plasma levels may reflect the actual status of the omega-3 PUFAs that should always be considered with respect to their effects.

## 3. Mechanisms and Factors Involved in the Development of VF and AF

In order to better understand the anti-arrhythmic potential of omega-3 PUFAs, we will briefly summarize the main mechanisms and key factors involved in arrhythmogenesis. The heart can “die” due to three major events: electromechanical dissociation, asystole and heart block, and VF [24]. The last event is the most frequent. Basic electrophysiological mechanisms of cardiac arrhythmias include abnormal electrical impulse generation, i.e., increased automaticity or triggered activity, and abnormal electrical impulse propagation, i.e., block of conduction and re-entry, whereby the simultaneous operation of both abnormalities occurs [24].

There are three main factors involved in the development of severe arrhythmias: arrhythmogenic substrates, triggers and modulating elements [25]. Arrhythmogenic substrates, such as myocardial structural and ion channels remodeling, in the setting of inflammation and oxidative stress, facilitate occurrences of VF and AF as well [26,27]. Abnormal calcium handling and high intracellular calcium as well as acidosis (usually ischemia-related) may all act as triggers [28,29,30] and alterations in autonomic tone are considered modulating elements [31]. The triggered impulses may result from early or delayed after-depolarization. The slowing or blocking of conduction is a consequence of changes in passive and active membrane properties [25]. These include changes in membrane composition, dysfunction of intercellular connexin (Cx) channel communication (uncoupling), suppression of sodium channel-dependent current and accumulation of extracellular matrix proteins [30,32].

Although we know the basic mechanisms that can cause arrhythmias, we have little understanding of the changes in cardiac electrical properties. These properties are the immediate cause of the operation of one or another of the arrhythmogenic mechanisms in various heart conditions or cardiac disease. In normal hearts, atrial flutter or AF is usually self-terminating [32] and transient VF is often observed in animals and sometimes even in humans [33].

Nevertheless, both AF and VF are assumed to occur due to abnormal impulse initiation and circuit movement re-entry. In the case of AF, the cardiomyocyte sleeves that extend into the pulmonary veins are a prime source of ectopic electrical activity, while re-entry circuits are probably promoted by atrial tissue heterogeneity (resulting in dispersion of repolarization) and by alterations in intercellular coupling, mediated by Cx channels [27,34]. In the case of VF, sympatho-vagal imbalance and abnormal calcium handling may trigger ectopic activity, while re-entry is facilitated by slowing and blocking the conduction [31,32]. Mechanistically, conduction slowing can be caused by reduced membrane excitability, reduced Cx channel mediation of cell-to-cell coupling and discontinuous tissue architecture [26].

In this context, the role of direct intercellular communication at the cardiac gap junction Cx channels should be emphasized. These nonselective intercellular channels ensure electrical and molecular signal propagation and contribute to the determination of conduction velocity [35]. Down-regulation and abnormal cellular distribution of the major gap junction protein, Cx43, as well as cell-to-cell uncoupling due to channel dysfunction have a highly arrhythmogenic effect, by promoting triggered activity, dispersion of repolarization and refractoriness and slowing and blocking conduction [36,37,38,39,40].

It has been reported that an over-expression of miRNA-1 inhibits electrical coupling and depolarized cardiac cell membrane, by post-transcriptional repression of Cx43-related, *GJA1*, and potassium related, *KCNJ2*, genes, which likely accounts for its arrhythmogenic potential [41]. The pro-arrhythmogenic effect of elevated miR-1 in the infarcted rat heart was attenuated by its down-regulation induced with propranolol [42]. However, investigations of miRNAs expression in human myocardium, in regard to arrhythmogenesis, are still rare [43].

In the context of arrhythmias, the central role of mitochondrial respiration in cardiomyocyte function should be noted. Numerous pathophysiological processes implicated in the development of AF and VF have been linked with mitochondrial dysfunction, including altered calcium homeostasis, excess reactive oxygen species (ROS) formation and alterations in oxygen consumption. Mitochondria are considered to be a source of metabolic sink and arrhythmias [44] as well as the targets for suppressing arrhythmias [45].

Despite progress in the treatment of heart diseases, SCD (due to infarction and VF) still remains a major cause of mortality worldwide. Although sophisticated devices, such as ICD, are efficient in preventing death due to VF when it occurs, they do not prevent its development and/or recurrence. This topic is still open to investigation and reduce the risk of the incidence of VF.

Another “life-threatening” arrhythmia, which has high risks of tromboembolism and stroke, is AF, whose incidence is increasing. Anti-arrhythmic drug therapies are often ineffective in terminating AF or preventing a recurrence of AF, possibly because these drugs target a single pathophysiological mechanism. Invasive approaches, using catheter ablation of arrhythmogenic triggers, do not prevent the recurrence of AF, likely because of the persistence of arrhythmogenic substrates and non-pulmonary vein triggers [46]. In advanced forms of AF, abnormal atrial substrates, including Cx43, Cx40 and Cx45 abnormalities, are thought to act as drivers of arrhythmia perpetuation. It seems that a better understanding of the risk factors for AF could initially prevent its occurrence or subsequently, more efficiently treat patients with AF [47,48,49].

Importantly, another clinical issue that should be addressed is post-operative AF (POAF), a common complication after cardiac surgery (accounting for up to 60% of patients). POAF is associated with an increased risk of cardiovascular mortality, stroke and other arrhythmias that can impact on early and long term clinical outcomes and health economics. Many factors, such as disease-induced cardiac remodeling, operative trauma, changes in atria pressure and sympathetic/parasympathetic activation, have been implicated in the development of POAF [50]. Moreover, inflammation, oxidative stress and transient ischemia-reperfusion may be involved in the pathogenesis of POAF [27].

It is of interest to note that the drugs having the most significant impact on severe arrhythmias are those without direct electrophysiological actions on myocardial excitable tissue [24]. However, these actions are yet to be explored. Such drugs are angiotensin-converting enzyme inhibitors, angiotensin receptor blockers, lipid lowering statins and aldosterone antagonists as well as non-pharmacological compound, omega-3 PUFAs. Thus, it appears that these drugs probably act via upstream effects on myocardial substrates, triggers or modulating factors, resulting in preservation of myocardial electrical stability [31]. Besides their impact on the occurrence of VF, these agents have been observed efficient to suppress AF as well [47,51]. It has been suggested that the anti-arrhythmic efficacy of omega-3 PUFAs may interfere with the anti-arrhythmic actions of these drugs.

Progress in treating cardiac arrhythmias includes diverse areas, such as: autonomic manipulation, drugs, imaging, devices, and genetics, suggesting the complexity in treating arrhythmias [31]. Because of this complexity, there is still a need to explore novel, multi-targeted, reliable approaches to prevent VF as well as AF; omega-3 PUFAs are a promising candidate.

## 4. Potential Targets of Omega-3 PUFAs Relevant to Arrhythmias Prevention

Advances in the molecular targets of omega-3 PUFAs associated with CVD have been recently reviewed [52,53], including G protein-coupled receptors, modulation of gene expression and transcription, production of novel anti-inflammatory lipid mediators, lipoxidative processes, modulation of the release of free radical species and prevention of structural remodeling. Omega-3 PUFAs can also have an impact on the prevention of thrombus formation and thromboembolism in patients suffering from AF, via the suppression of factor VII activating protease [54]. Thus, the beneficial effects of omega-3 PUFAs can be attributed to a synergism between multiple mechanisms of action, whereby there is a close association between the effect and function [55].

The available literature suggests that the anti-arrhythmic effects attributed to omega-3 PUFAs include direct and indirect modulation of ion channel properties, membrane composition and fluidity as well as anti-inflammatory and anti-fibrotic effects and modulation of sympatho-vagal balance [56]. Direct inhibition of sarcolemmal ion channels (Na^+^, Ca^2+^ and various K^+^ currents) in some pathological conditions may stabilize electrical activity and prolong the relative refractory period of the cardiomyocytes. These omega-3 PUFA properties resemble actions of anti-arrhythmic drugs—class I, class II and class III—according to the older classification. However, unlike these drugs, omega-3 PUFAs are rarely proarrhythmic. A modulation of intracellular calcium handling by omega-3 PUFAs, including inhibition of sarcoplasmic reticulum RyR channels, and prevention of calcium overload may abolish or attenuate arrhythmia triggers [57,58,59].

While circulating or free omega-3 PUFAs may directly affect ion channel properties, the incorporated omega-3 PUFAs in the cardiomyocyte membranes affect their fluidity and hence allosteric structure, and thereby the function of ion channels. This may contribute to the attenuation of myocardial electrical instability, an improvement in myocardial tissue function [53,57,60] and a termination of asynchronous contractile activity [61]. Lowering the cardiomyocyte excitability can be particularly appreciated within ischemic myocardial tissue, which is more susceptible to partial depolarization and triggered arrhythmias [62]. However, it is unknown whether circulating omega-3 PUFAs enhance or diminish the effects of incorporated omega-3 PUFAs. In rabbit cardiomyocytes, the action potential shortening, induced by circulating omega-3 PUFAs, was prevented by incorporated omega-3 PUFAs, which does not suggest a cumulative effect [63].

Long-term omega-3 PUFA supplementation in humans has prolonged atrial refractoriness and reduced vulnerability to inducible AF [64], which may explain their anti-arrhythmic effects. However, acute infusion of omega-3 PUFAs did not alter atrial refractory periods, but resulted in transient atrial conduction slowing, suppression of AF inducibility and conversion of AF into atrial flutter [65]. Interestingly, the atrial flutter inducibility was enhanced in these patients, without structural changes to the heart, suggesting that a high dose of intravenous infusion may be proarrhythmic in some circumstances.

Available findings suggest that omega-3 PUFAs, via improvements in autonomic function, endothelial function, lowering of blood pressure, anti-inflammatory actions, modulation of the blood lipid profile, reduction of oxidative stress and calcium overload as well as maladaptive myocardial remodeling (fibrosis) [66,67] may suppress the development of arrhythmia substrates, and the occurrence of arrhythmia triggers and pro-arrhythmic modulators.

DHA is recognized as a potent anti-arrhythmic agent. A recent article [68] summarized current knowledge on the metabolic pathways involved in the generation of biologically active DHA metabolites, such as resolvins, protectins, maresins, neuroprostanes and others. They have been shown to exert many beneficial actions, including anti-arrhythmic effects. Indeed, in an in vivo post-myocardial infarction mouse model, oxidized DHA and 4(RS)-4-F4t NeuroProstane prevented arrhythmias and posttranslational modifications of RyR2 [69]. This suggests that products of non-enzymatic oxidized omega-3 PUFAs may attenuate RyR complex destabilization during ischemic events. Thus, novel bioactive derivatives of DHA appear to be agents that could potentially be used in clinics.

In addition, the membrane-incorporated DHA has been reported to inhibit cardiac adrenergic stimulation [70], which is associated with ischemia and HF. DHA is also effective in reducing factors that perpetuate AF [70], including wall stretch and relative ischemia.

The pro-inflammatory cytokine, interleukin-1β, which is increased in the heart, post-AMI, has been shown to cause the loss of Cx43 function, which underlies formation of the arrhythmogenic substrate. The omega-3 PUFA treatment inhibited interleukin-1β-stimulated loss of Cx43 protein, by inhibiting the translocation of the nuclear factor NF-κB [71].

An experimental study, using rabbits [72], demonstrated the anti-arrhythmic effect of EPA, which dose-dependently reduced the pulmonary vein spontaneous beating rate and the amplitude of delayed after-depolarizations, via mechano-electrical feedback, generated by NO production. Long-term treatment with EPA also ameliorated post-ischemic reperfusion-induced injury, partly through myocardial Rho-kinase pathway inhibition and preservation of eNOS activity in an in vivo pig model [73].

Dietary omega-3 PUFAs suppressed the up-regulation of sodium-hydrogen antiporter 1 activity (known to be enhanced during ischemia/reperfusion, hypertrophy and HF) and lowered the incidence of triggered activity, in a rabbit model, of volume and pressure overload [74].

It is important to emphasize that omega-3 PUFAs are involved in the modulation of various mitochondrial processes, including mitochondrial calcium homeostasis, gene expression, respiratory function, ROS production and mitochondrial apoptosis. Therefore, mitochondria seem to play a central role in the mechanisms underlying the protective effects of PUFAs [44,75]. In support of this, there are findings that show that EPA and DHA compete with arachidonic acid for the conversion by cytochrome P450 (CYP) enzymes, resulting in the formation of novel epoxy and hydroxy metabolites that are physiologically active [76]. Dietary EPA/DHA supplementation has caused a profound shift in cardiac CYP-eicosanoid profiles, from arachidonic acid to EPA and DHA derived epoxy- and hydroxy metabolites, which exert highly-potent anti-arrhythmic properties. CYP-eicosanoid mediated mechanisms are beneficial in cardiac ischemia–reperfusion injury and maladaptive cardiac hypertrophy [77].

Omega-3 PUFAs supplementation has also been shown to affect gene expression. Many of the down-regulated genes in rat cardiomyocytes appear to be related to inflammation, extracellular matrix remodeling, calcium handling and ROS generation [78]. The modulation of specific genes by omega-3 PUFAs and cross-talk between these genes are thought to be responsible for many effects of omega-3 PUFAs [79]. These findings allow the assumption that some anti-arrhythmic effects of omega-3 PUFAs are due to direct modulation of genes in cardiomyocytes.

DHA and EPA, infused in spontaneously beating isolated rabbit hearts, have dose-dependently increased the threshold for elicitation of a ventricular extrasystole and DHA has reduced longitudinal propagation velocity [80]. The latter might be associated with the modulation of intercellular gap junction Cx43 channel conductivity. This mechanism is likely involved in the acute anti-fibrillating and defibrillating effects of EPA and DHA, demonstrated in old, hereditary hypertriglyceridemc rats [81]. However, this needs to be proven.

Dietary omega-3 PUFAs have been shown to improve electrical remodeling in a rat model of human high rennin hypertension [82]. Prolonged QRS and QTc intervals and increased T-wave dispersion were reduced by omega-3 PUFAs or aliskiren. Both treatments reduced arrhythmia induction, macrophage infiltration and fibrosis as well as restored normal topology of Cx43. Experimental studies using various animal models imitating human diseases, such as hereditary hypetriglyceridemic rats, spontaneously hypertensive rats and diabetic rats, revealed benefits from long-term omega-3 PUFA intake. This resulted in a reduced incidence of electrically induced VF [22,23,83] or improvement of cardiac function (ejection fraction) in diabetic rats [84]. These effects were associated with the up-regulation of myocardial Cx43 and attenuated its abnormal cardiomyocyte distribution, likely via protein kinase C epsilon (PKCε)- mediated signaling pathways. It appears that cardiac Cx43 may be a promising target for the prevention of malignant ventricular arrhythmias. In addition, it has been reported recently that treatment with omega-3 PUFA and antioxidant vitamins reduces oxidative and nitrosative stress and prevents Cx40/Cx43 lateralization in human atrial tissue, which likely contributes to POAF prevention. However, it failed to fully prevent POAF occurrence, due to the fact that these compounds did not normalize Cx40 down-regulation and Cx45 up-regulation, that may promote POAF [85].

Taken together, it appears that the biological and specific anti-arrhythmic potentials of omega-3 PUFAs must be a well-coordinated mechanism. Previously established and novel omega-3 PUFAs mechanisms and targets may help to understand the role of omega-3 PUFAs in the protection of the heart against arrhythmias. Nevertheless, despite the above-mentioned evidence, there is still a need to explore direct cause-effect relationships and to characterize more exactly the conditions in which omega-3 PUFAs are beneficial. This may facilitate translation of experimental data into clinics.

## 5. Anti-arrhythmic Efficacy of the Omega-3 PUFAs

This chapter provides data from experimental and clinical studies as well as meta-analyses published in the last decade, regarding anti-arrhythmic aspects of omega-3 PUFAs.

### 5.1. Omega-3 PUFAs and Prevention of AF and POAF

Once AF develops, drugs and invasive treatments, aimed at eliminating AF and maintaining sinus rhythm have significant risks, limited long-term success rates, and do not reduce adverse outcomes associated with AF [47,48]. There is a need to look for novel, safer therapies, to treat established AF and to prevent persistence of AF as well as preventing undesirable POAF. Taking into consideration the wide spectrum of effects provided by omega-3 PUFAs (see chapter IV) and almost no adverse effects, one would expect them to induce a prophylactic or treatment benefit.

The red blood cell membrane fatty acid composition in patients, with and without AF, revealed surprisingly significantly higher percentages of total omega-3 PUFAs in AF patients, than in controls [16]. The question arises of whether AF itself may activate self-mechanisms to fight arrhythmias via increased membrane omega-3 PUFAs and decrease undesirable saturated and trans-fatty acids.

It is interesting to note that metoprolol, like omega-3 PUFAs, may affect AF occurrence, via the suppression of abnormalities in gap junction Cx43 and Cx40 distribution and atrial conduction in human AF patients [86]. These findings point out the novel pleiotropic anti-arrhythmic mechanisms of metoprolol. It appears that the anti-arrhythmic effect of beta-adrenergic blockers (often used in heart disease patients) may interfere with the anti-arrhythmic effect of omega-3 PUFAs. An attenuation of an abnormal Cx43 distribution by omega-3 PUFAs resulted in a reduction in POAF incidence [85].

DHA and EPA supplementation increased their levels in plasma and atrial tissue phospholipids in dogs [70]. DHA (1 g/day orally for 21 days) was shown to be more effective than EPA in attenuating AF vulnerability and atrial remodeling (fibrosis), in pacing-induced AF in dogs. The increases in atrial systolic and diastolic volumes, caused by pacing, were significantly smaller in the DHA group, while not affected by EPA, compared to the control group. These findings suggest an inverse relationship between atrial tissue DHA content and vulnerability to AF.

Omega-3 PUFAs supplementation (2 g/day, Omacor EPA and DHA 1.2:1), 4 weeks before intervention-attenuated inducibility and maintenance of AF in a sterile pericarditis dog model—induced by reducing pro-inflammatory cytokines—prolonged an effective refractory period and shortened conduction time [87]. In a rabbit study [88], a DHA-enriched diet resulted in lower AF inducibility and duration.

An investigation into the effects of a new pure DHA derivative called F 16915, in experimental rat and dog models of HF-induced atrial dysfunction, showed that F 16915 (5 g/day for 4 weeks) significantly reduced the incidence of sustained AF in the dog model and also reduced the non-functional dephosphorylated form of Cx43 in rat atrial tissue [89]. The findings suggested that F 16915 might be a promising new drug as an upstream therapy for the treatment of AF in patients with HF. The incidence of AF was decreased after treatment with omega-3 PUFAs (1.2 g/day, EPA and DHA 2:1, for 14 days), which increased atrial tissue omega-3 PUFAs levels in a dog model of vagally-induced AF [90]. This protection was mostly related to a decrease in elevated total and phosphorylated Cx40 expression.

Serum DHA, but not EPA, was associated with a lower inducibility of AF in a study of patients without AF and without structural heart disease [65]. A low DHA (0.2–0.9% vs. 1.4–3.1%) was associated with atrial dilatation (which predisposes AF) in patients with HF [91]. Dilatation was identified as a target for 1–2 g of omega-3 PUFAs/day.

Plasma DHA, but not EPA, was correlated with a lower AF incidence in a cohort of more than 3000 elderly Americans [92]. Serum DHA, but not EPA, was significantly inversely correlated with risk of AF in an ischemic heart disease risk factor study [93]. It appears that it is challenging to explore the specific effects of EPA and DHA on signaling pathways and biological processes underlying antiarrhythmic benefits in humans as well as in experimental animal models. Unfortunately, incorporated RBC levels of DHA and EPA were not registered to allow the determination of any relationships between circulating vs. tissue levels of EPA and DHA.

The most recent meta-analysis revealed a U-shaped relationship between red blood cell DHA and POAF [94]. The DHA range of 7.0–7.9%, had the lowest incidence of POAF. Subjects in the lowest, but also highest, levels had a significantly higher risk of developing POAF. The possibility of an increased risk of POAF at high levels of DHA suggests an upper limit for omega-3 PUFAs in certain conditions. In the Diet, Cancer and Health Cohort Study, a total of 57,053 Danish participants, 50–64 years of age, were enrolled. A U-shaped association between consumption of omega-3 PUFAs and the risk of incident AF was found, with the lowest risk being when intake was 0.63 g/day [95].

An inverse association was found between the presence of AF and the plasma DHA in haemodialysis patients with CVD, treated with omega-3 PUFAs (1.7 g/day) for 3 months [96]. The recurrence of AF after electrical cardioversion was reduced in patients with persistent AF, using amiodarone and a renin–angiotensin–aldosterone system inhibitor, in addition to omega-3 PUFAs (2 g/day, Omacor, EPA and DHA 1.2:1) during a 1-year follow-up [97]. Likewise, long-term (>1 month) supplementation with omega-3 PUFAs (6 g/day, DHA 1.5 g, EPA 0.3 g) prior to electrical cardioversion reduced the recurrence of AF [98] and attenuated atrial mechanical stunning after reversion of AF into sinus rhythm [65].

Results from the prospective randomized study on the effect of long-term omega-3 PUFA supplementation on the paroxysmal atrial tachyarrhythmia burden in patients with implanted pacemakers have shown that there is no suppression of the AT/AF burden but its temporal progression, related to aging and sinus node disease may be attenuated [99].

Dietary fat intake is differentially associated with the risks of paroxysmal compared with sustained AF, in women. A prospective cohort study in 33,665 women aged >45 years, without CVD or AF at baseline, was performed [100]. High saturated fatty acids and low monounsaturated fatty acids intakes were associated with a greater risk of persistent or chronic, but not paroxysmal, AF. Improving dietary fat quality may play a role in the prevention of sustained forms of AF.

Omega-3 PUFA administration (2 g/day, EPA/DHA 1:2) for at least 5 days before surgery) significantly reduced the incidence of POAF in 96 patients undergoing “on-pump” coronary artery bypass graft surgery [101]. The same approach reduced the incidence of POAF in patients with a recent (≤3 months) myocardial infarction, who were undergoing cardiac surgery [102]. In addition, patients in the treatment group had significant shorter intensive care unit stays and suffered less frequently from impaired wound healing. The reduced incidence of POAF and shorter stay in hospital was achieved by pre- and peri-operative intravenous infusion of omega-3 PUFA (Omegaven^®^ 100 mg/kg body weight/day, Fresenius SE & Co. KGaA, Bad Homburg, Germany), starting at admission to hospital and ending at discharge from intensive care [103].

In addition, a recent meta-analysis suggested that preoperative administration of omega-3 PUFAs significantly prevented the occurrence of POAF in patients undergoing cardiac surgery [104,105]. The combination of omega-3 PUFAs (2 g/day, EPA/DHA 1:2) with vitamins C and E was more efficient at increasing the antioxidant potential and attenuating oxidative stress and inflammation [106]. The ratio of EPA/DHA may influence the incidence of POAF, and 1:2 may be the most appropriate ratio [107].

The preoperative levels of EPA and arachidonic acid in plasma phospholipids and red blood cell membrane lipids were associated with changes in pro-inflammatory and anti-inflammatory mediators, suggesting a complex role in the postoperative inflammatory process [108]. This evidence could explain, at least in part, discrepancies in omega-3 PUFA efficacy in preventing POAF.

Besides the beneficial effects of omega-3 PUFAs in the context of AF, there some recent studies failed to demonstrate this benefit. The treatment with vitamins (500 mg ascorbic acid and 45 IE vitamin E) and omega-3 PUFAs (two pre-operative infusions of Omegaven^®^, EPA and DHA ~0.15 g/kg and a third infusion dose of 5 g, applied 42 h after surgery) attenuated post-operative oxidative stress during the course of a cardiac artery bypass graft surgery, but was inefficient with respect to POAF onset and its duration [109]. Another study showed that pre-operative EPA administration resulted in suppression of post-operative inflammation (possibly through an increase in plasma adiponectin levels) but it did not significantly prevent POAF [110]. Treatment with EPA (1.8 g/day for 6 months), in combination with anti-arrhythmic drugs, did not significantly reduce either the AF burden or the CRP levels in paroxysmal AF patients who had no evidence of substantial structural heart disease [111]. These findings point out that the anti-arrhythmic potential of EPA is much less pronounced when comparing to DHA.

A high dose (4 g/day) of omega-3 PUFA, added to conventional therapy in patients with paroxysmal or persistent AF, did not reduce its recurrence or markers of inflammation and oxidative stress [112]. It is possible that concomitant therapy with drugs, that may suppress arrhythmogenesis via pleiotropic properties, could interfere with the effects of omega-3 PUFAs.

A randomized trial of omega-3 PUFA infusion to prevent POAF failed to prevent the occurrence of AF after cardiac surgery during a 2-year follow-up period [113]. However, neither O3I, nor plasma pre-interventional levels of omega-3 PUFAs, were registered. Thus, it is difficult to explain the effect of infusion of high doses of omega-3 PUFAs (200 mg/kg/day starting before anesthesia for 24 h, followed by 100 mg/kg/day for 7 days) in thirty-nine patients suffering from CHD.

Supplementation with 1 g/day of the omega-3 PUFAs, for 1 year, did not reduce recurrent AF in patients with symptomatic paroxysmal AF that required cardioversion [114]. This prospective, randomized, double-blind, placebo-controlled, multicenter FORVARD trial involved 586 outpatients—men and women. The power of this trial seems to be affected by the heterogeneous population of patients, suffering from various systemic or heart diseases and who likely had different omega-3 PUFA status’s. Similarly, the AFFORD trial [115] showed that a high dose of omega-3 PUFAs did not reduce AF recurrence in patients with a history of AF, who were not receiving conventional anti-arrhythmic therapy. Furthermore, the treatment did not reduce inflammation or oxidative stress markers in this population that may explain the lack of omega-3 PUFAs efficacy; however, there are questions over why this occurred.

Accordingly, recent reviews [56,116] and meta-analyses [117,118] have concluded that nowadays, there are no sufficient data to encourage omega-3 PUFA therapy in AF patients. It is suggested that a single dose-ranging phase II trial is mandatory to establish the optimal dose. Only a big phase III trial with good statistical power, a hard, primary end point and a real-life population would allow us to make up our mind regarding this interesting issue.

Likewise, a meta-analysis of four randomized studies, involving 538 patients, published in 2011, concluded that there was insufficient evidence to suggest that treatment with omega-3 PUFAs reduces POAF. Therefore, their routine use in patients undergoing cardiac surgery is not recommended [119,120].

However, as noted in these meta-analyses, the lack of statistical power and the heterogeneity among studies could, in part, explained the lack of benefit. Moreover, it is necessary to strengthen the idea that neither original trials, nor meta-analyses took into consideration the importance of basal omega-3 PUFA status monitoring, i.e., both plasma and O3I. Thus, it seems that the relevance of the conclusions of clinical trials is difficult to justify.

### 5.2. Omega-3 PUFAs and Prevention of Malignant Ventricular Arrhythmias

After identifying a wide spectrum of direct or indirect anti-arrhythmic effects of omega-3 PUFAs (see chapter IV), as well as the prevention of AF, it is of interest to explore their potential for decreasing the risk of the development of life-threatening ventricular arrhythmias, that also affect quality of life in patients with ICD.

A low value for DHA was associated with greater left ventricular dilatation (which promotes the risk of malignant arrhythmias), while a higher level had a 91% negative predictive value for severe dilatation. Based on administration of the omega-3 PUFAs, dilatation was identified as a target for 1–2 g omega-3 PUFAs/day [91].

It has been reported that a low serum EPA level is accompanied by the occurrence of J-waves and higher incidence of ventricular tachycardia (VT)/ventricular fibrillation (VF) after AMI [66,121]. This suggests involvement of the J-wave and its possible link with EPA in the prediction of ischemia-induced VT/VF in these patients. Early EPA treatment after percutaneous coronary intervention reduced acute inflammatory responses and ventricular arrhythmias in patients with AMI. In addition, low serum levels of omega-3 PUFAs (EPA + DHA < 155 mg/mL) before percutaneous coronary intervention may be a predictor of reperfusion-induced ventricular arrhythmias [122].

In a small, randomized, placebo-controlled cross-over study, patients with an ICD, who underwent electrophysiological testing and received an intravenous infusion of 3.9 g omega-3 PUFAs tended to have decreased VT inducibility compared to a placebo group [123]. Therefore, a larger study is warranted.

The most recent study [124] indicated that among 87 patients with ischemic cardio-myopathy and ICD, the number of VT episodes was significantly lower following treatment with omega-3 PUFAs (3.6 g of EPA and DHA for 6 months). Their results suggest that omega-3 PUFAs supplementation may be associated with a reduction in the frequency of tachyarrhythmias, and hence electrical shocks, in ICD recipients with CHD.

Findings from an experimental study [125] demonstrated that ischemia-induced VF was significantly reduced in male pigs fed with EPA (600 mg/kg/day) for 3 weeks and and subjected to myocardial ischemia for 90 min, in vivo. EPA significantly inhibited the expression of Kir6.2, a major component of sarcolemmal K (ATP) channels, in the ischemic region; this may explain the prevention of (pro-arrhythmogenic) action potential shortening. The propensity of saturated fat fed rats to postichemic reperfusion-induced arrhythmias, was reduced due to omega-3 PUFA supplementation [126]. A dietary supplement dose, as low as 3% of total fat, effectively reversed the high oxygen requirements.

Supplementation with omega-3 PUFAs (EPA/DHA 1.2:1, 20 mg/day for 2 months), in old male and female spontaneously-hypertensive rats (the rat strain with reduced O3I, [21]) resulted in multiple cardioprotective effects, such as a significant decline in blood pressure, suppression of inducible VF by 57% (male) and 67% (female), up-regulation of Cx43, attenuation of arrhythmogenic substrates (fibrosis and abnormal gap junction distribution), preservation of cardiac cell membrane integrity, enhancement of energetic metabolism and augmentation of myocardial capillary density [22]. Omega-3 PUFAs intake significantly reduced cardiovascular risk factors, suppressed inducible VF and facilitated sinus rhythm restoration in both young and old spontaneously-hypertensive rats [23]. The supplementation attenuated the mislocalization of Cx43, suppressed elevated Cx43 mRNA and enhanced expression of total Cx43 and/or its functional phosphorylated forms. Moreover, the omega-3 PUFAs diet enhanced the expression of cardioprotective PKCε and suppressed the proapoptotic PKCδ isoform as well as normalized contractile proteins (MyHC) profiles, at the early stage of the disease. These results support the prophylactic use of omega-3 PUFAs to minimize cardiovascular risk and sudden arrhythmic death in individuals suffering from hypertension.

Omega-3 PUFA ethyl-esters (25 g/kg diet) or a direct renin inhibitor (aliskiren; 3 mg/kg/day) reduced arrhythmia induction, from 75% to 17%, vs. 0% in rats suffering from high renin-induced hypertension [82]. This was associated with an attenuation of prolonged QRS and QTc intervals and increased T-wave dispersion as well as abnormal Cx43 expression.

Another study demonstrated that the treatment of hereditary hypertriglyceridemic rats with omega-3 PUFAs (30 mg/100 g body weight) or atorvastatin (0.5 mg/100 g body weight), for 2 months, increased the threshold for inducing VF [83]. In addition, both treatments attenuated abnormal myocardial Cx43 distribution (lateralization and internalization) and decreased hyper-phosphorylation of Cx43. As both compounds are ligands for peroxisome proliferator-activated nuclear receptors, it is of interest to explore the pathways that may be involved in these beneficial effects. In addition, acute anti-fibrillating and defibrillating potentials of EPA, DHA and atorvastatin were demonstrated, ex vivo, using isolated hearts from old male and female hereditary hypertriglyceridemic rats [81]. Sustained VF was electrically induced in all untreated rat hearts. In contrast, the induction was significantly reduced by EPA and DHA as well as by atorvastatin (all at a dose of 15 μmol) in male and female rat hearts. Moreover, a bolus (150 μmol) of EPA and DHA, administered directly to the fibrillating heart restored sinus rhythm in six out of six hearts and atorvastatin in four out of six hearts. These findings point out the clear cut, acute, anti-fibrillating efficacy of examined agents and challenge us to explore the underlying mechanisms.

The anti-arrhythmic effect of free omega-3 PUFAs was observed in an experimental LQT2 and LQT3 rabbit model, due to the suppression of early after-depolarization and reduction of spatial and temporal dispersion of repolarization. The VT preventive effect was remarkable for both DHA and EPA [127].

When a diet low in saturated fats and omega-6 PUFA, but rich in omega-3 PUFAs, was applied in rats, it resulted in smaller myocardial infarct sizes that may reduce SCD [128]. Moreover, there was an accumulation of omega-3 PUFAs and a decrease in arachidonic acid in plasma, cardiac cell membranes and mitochondria. It is suggested that increased tolerance to ischemia-reperfusion injury may be one of the critical factors explaining the protective effects of dietary omega-3 PUFAs, which are associated with CHD complications in humans.

Rats supplemented for a period of 12 weeks, with either echium oil or tuna oil (high in DHA) showed a dose-related elevation in DHA: 14.8–24.1% of total fatty acids. At the highest dose level, tuna oil rats displayed significantly lower episodes of ischemia induced VF (29% vs. 73%) [129].

A meta-analysis of randomized trials, conducted on 32,919 patients, showed that when comparing omega-3 PUFAs to a placebo, there is a non-significant risk reduction for SCD and ventricular arrhythmias [130]. However, when evaluating the significance of omega-3 PUFAs efficacy it is necessary to take into consideration the omega-3 PUFA endogenous status and drugs regimen in patients, to prevent false negative conclusions.

Dietary omega-3 PUFAs (1–4 g/day) for 3 months significantly increased their levels in red blood cell and left ventricular tissue in dogs. However, despite large (or probably because too large) increases in cardiac tissue omega-3 PUFA contents, this did not prevent ischemia-induced VF and actually increased arrhythmia susceptibility in non-infarcted dogs as well as low-risk post-MI dogs [131]. There were some limitations in this study, e.g., the dose-dependent effect on the risk of VF in respect to the pre-ischemic levels of omega-3 PUFAs was not evaluated. This might explain null or even pro-arrhythmia events. Moreover, this healthy-heart dog model seems to not be relevant to clinical conditions because VF incidence (and the more AMI) is rare in healthy individuals without cardiovascular risk factors or genetically-related heart diseases. These limitations should be taken into consideration when interpreting the findings obtained in the same canine SCD model [132,133]. Another study demonstrated that supplementation of rats with omega-3 PUFAs for 3 weeks did not protect against AMI related VT/VF, although, at the cellular level they prevented calcium overload [134]. All limitations of the study should be taken into consideration when analyzing the possible factors implicated in the lack of anti-arrhythmic effects.

Taken together, recent data indicate that long-term omega-3 PUFA supplementation suppresses the mechanisms and factors involved in the development of life-threatening ventricular arrhythmias and has the potential to decrease a number of electrical shocks in patients with ICD. Further, well designed experimental studies, mimicking clinical conditions are needed, to get a more comprehensive view on the molecular pathways as well as intra- and intercellular signaling implicated in the anti-arrhythmic effects of omega-3 PUFAs. No less important, is determining the conditions and exploring the mechanisms of adverse and eventually pro-arrhythmic actions of omega-3 PUFAs.

As previously noted [135], if omega-3 PUFAs do protect against SCD in the setting of ischemia, or in structurally remodeled hearts, the public health impact, in terms of quantity and quality of lives saved, has the potential to be large. Therefore, the benefits and risks of omega-3 PUFAs both require and deserve to be adequately tested. Moreover, the role of O3I in predicting life-threatening arrhythmias should be tested in both clinical and experimental conditions, via the monitoring of RBC or tissue-incorporated omega-3 PUFA status.

## 6. Conclusions

Recently published papers included in this review suggest that there is a permanent interest of both clinical and experimental cardiologists, to explore the anti-arrhythmic potential of omega-3 PUFAs. Although apparent progress is observed and the majority of findings suggest benefits of omega-3 PUFAs, there is still not enough evidence for their routine implementation into the clinic. Nevertheless, criticisms and limitations of the trials as well as novel experimental findings suggest the need for further investigations. There is no doubt that the efficacy of omega-3 PUFAs should always be related to the basal pre-interventional omega-3 PUFAs status, which is determined by the omega-3 index and plasma omega-3 PUFA levels of participants. This may promote more objective evaluations and convincing findings, in regard to the anti-arrhythmic efficacy of omega-3 PUFAs, in the near future.
